# Siphonophore genome structure and the evolution of functional specialization

**DOI:** 10.1371/journal.pone.0351247

**Published:** 2026-07-02

**Authors:** Namrata Ahuja, Darrin T. Schultz, Dalila Destanović, Samuel H. Church, Natasha Picciani, Catriona Munro, Koto Kon-Nanjo, Tetsuo Kon, Maciej K. Mańko, Wendy Shi, Oleg Simakov, Casey W. Dunn

**Affiliations:** 1 Department of Ecology and Evolutionary Biology, Yale University, New Haven, United States of America; 2 Department of Neurosciences and Developmental Biology, University of Vienna, Vienna, Austria; 3 CNRS, Laboratoire de Biologie du Développement de Villefranche-sur-mer (LBDV), Sorbonne Université, Villefranche-sur-Mer, France; 4 Department of Marine Biology and Biotechnology, Laboratory of Plankton Biology, University of Gdańsk, Gdynia, Poland; Tsinghua University, CHINA

## Abstract

Siphonophores (Cnidaria: Hydrozoa) are pelagic colonial marine invertebrates with many highly specialized bodies (zooids) within a single colony. Their unique biology and ecological importance have made them of particular interest, and motivate questions on their genomic structure, organization and content. To investigate siphonophores’ genome biology and develop resources for future studies, we sequenced the genome of a single *Nanomia septata* to chromosome scale. The haploid genome is 1.7 GB across 8 chromosomes. Relative to closely related hydrozoan genomes, this is an expansion in length but a reduction, from 15, in chromosome number, indicating multiple chromosomal fusion events. We found no genomic features clearly associated with siphonophores’ exceptional colony-level complexity. Gene families that play critical roles in cnidarian development have not expanded, and gene proximity was not generally correlated to their expression across zooids, except in male gonophores. To contextualize these observations, we sequenced 20 additional *Nanomia* specimens across the globe and mapped them to our chromosome-scale reference. Population genomic analyses support three previously recognized species of *Nanomia*, and at least one additional undescribed species. Present day overlapping geographic distribution of some *Nanomia* species raises the possibility of reproductive isolation in sympatry. Phylogenetic analyses of genome size indicate *Nanomia septata* and *Nanomia cara* have similarly large genomes around 1.7 GB, while *Nanomia bijuga* and an undescribed species show a secondary reduction to 0.7 GB. These results highlight how genomic factors have shaped colony organization and genome diversity within *Nanomia.*

## Introduction

Siphonophores (Cnidaria: Hydrozoa) are animals with complex colony-level body plans [1, 2]. Though each siphonophore colony grows from a single embryo, they asexually produce many specialized bodies, or zooids, each with distinct functions and morphologies [1,3,4]. These bodies share resources through a common gastrovascular cavity and are highly physiologically integrated [1, 3]. This functional specialization of bodies [1, 3, 4] within a colony has long made siphonophores of central interest to questions about the evolution of new levels of biological organization, analogous to the transition from unicellularity to multicellularity or to the evolution of functional specialization of cells within a body [4–6].

**Fig 1:**
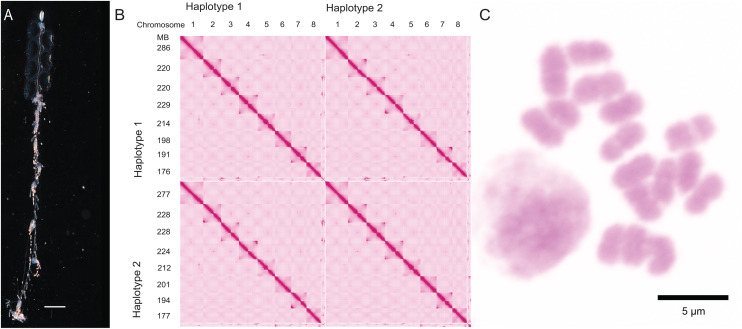
Genome of *Nanomia septata.* A. *Nanomia septata*. Scale bar is 1 cm. Photo credit to C. Munro. B. Omni-C Heatmap of genome assembly. Haplotype 1 is on the upper left, and haplotype 2 is on the lower right with the upper right and lower left squares representing the overlap between both haplotypes. Each chromosome is numbered 1–8 for each haplotype along the top row. Numbers on the left side give the approximate length of each chromosome in MB. C. *Nanomia septata* karyotype showing 2n = 16. A stained nucleus is present in the bottom left.

*Nanomia septata* (Fig 1A)*,* has emerged as a particularly useful model species for the study of these unique features of siphonophore biology. This species is abundant in coastal waters of the northeast Pacific, where it can be reliably collected [7]. Studies have described the budding process by which the colony grows [1], assessed differential gene expression across their zooids [8], examined the spatial patterning of i-cell genes within a colony [9], and characterized some of the cell types present in the colony [10]. Additionally, *Nanomia septata* have been cultured through multiple generations [11]. Further studies using *Nanomia septata* will advance what we know about siphonophore colony-level growth and functional specialization, including what mechanisms drive zooid differentiation and when zooids acquire their functional identity. These questions can only be answered with a high quality siphonophore genome.

Multiple cnidarian genomes have been sequenced to chromosome-scale in recent years. They tend to be relatively small for animal genomes, at 300–700MB [12–16], though larger cnidarian genomes have been found [17, 18]. Within Hydrozoa, siphonophores are most closely related to Filifera III + Filifera IV [19], a group that includes several well studied species including *Hydractinia symbiolongicarpus*, *Hydractinia echinata*, and *Podocoryna carnea*. Macrosynteny is highly conserved across many non-siphonophore cnidarians [20]*. Hydra vulgaris* and *Hydractinia symbiolongicarpus* form a paraphyletic grade with respect to siphonophores, with *Hydractinia symbiolongicarpus* being the closer relative to siphonophores [19]. High quality chromosome scale genome assemblies reveal that *Hydra vulgaris* and *Hydractinia symbiolongicarpus* have strikingly similar genome organizations, each with 15 chromosomes and similar gene content, despite there being about a two times size difference of 912 MB and 483 MB, respectively [12, 14, 20]. The conserved features of these two species, therefore, provide a clear picture of the ancestral genome structure, likely with 15 haploid chromosomes and a small genome, from which the genomes of siphonophores evolved.

It was surprising, then, when a new study on genome sizes of 35 siphonophores showed that many siphonophore genomes are larger than most other cnidarians [21]*.* Given the large genomes of siphonophores and their high degree of functional specialization of zooids, how is the complex biology of siphonophores reflected and associated with their genomes, and what can they tell us about siphonophore evolution? Are large genomes reflective of highly modular and many, distinct bodies within animal colonies? Have key gene families involved in zooid development expanded within siphonophore genomes? Are zooid specific genes expressed together in the spatial landscape of the genome? To understand how functional specialization has evolved, it is imperative to understand more about genome biology within siphonophores.

Of particular interest within the genome study of siphonophores is *Nanomia* since *Nanomia septata* had an estimated genome size around 1.4 GB, larger than typical of other cnidarians but within the range of siphonophore genome sizes, while *Nanomia bijuga* has a much smaller genome of only about 0.7 GB [21]. This previous work found that the *Nanomia septata* genome is about 63% repetitive [21], partially explaining the large size, though the types of repeats present were unknown. The genome differences between these two *Nanomia* species offer an opportunity to expand our understanding of population differences and diversity at the genomic level.

Only two species of *Nanomia*, *Nanomia bijuga* globally and *Nanomia cara* in the North Atlantic, were recognized as valid for most of the past century [22]. A new study based on morphology and sequences of two mitochondrial genes made critical progress toward understanding global *Nanomia* diversity [7]. It presented strong support for a distinct species in the North Pacific, which the authors describe as *Nanomia septata* [2, 7]. Before 2024, all specimens of *Nanomia septata* had been referred to as *Nanomia bijuga* in the literature (though not all specimens referred to as *Nanomia bijuga* are *Nanomia septata*). This previous study also found several *Nanomia* specimens that are likely from additional undescribed species [7]. Newer and improved methods and tools are well suited to advancing our rapidly changing picture of diversity of open ocean animal systems, including on *Nanomia*. Questions on genome biology and diversity are critical to using *Nanomia* as a model organism for the study of siphonophore biology, specifically those that address the evolution of zooid specialization and development, as well as addressing general questions of broad interest about the structure of animals in the open ocean.

## Results

### Genome description

#### Genome structure and annotation.

We present a highly contiguous, complete chromosome-scale diploid assembly of a single *Nanomia septata* colony collected near Monterey, California (Fig 1B). Each of the two haplotypes has 8 chromosomes (1794 and 1796 Mbp, respectively) and a BUSCO score of ~92% (Table 1 and Fig 1). A karyotype confirmed there are 2n = 16 chromosomes (Fig 1C). We designate each chromosome with a number 1–8. Haplotype 1 has 96.7% of the bases in 8 chromosomes, and haplotype 2 has 96.9% of bases in 8 chromosomes. The haplotypes are very similar to each other, with haplotype 2 having a slightly higher completeness and assembly quality (Table 1). Haplotype 2 also had fewer gaps than the alternate, and contained more contigs within the chromosome-scale scaffolds, resulting in fewer orphan scaffolds. Thus, we chose to use haplotype 2 for subsequent analyses, including annotation (Table 2). We created multiple genome annotations, including one using the *Nanomia septata* Iso-seq data. We found that the annotation made with the Iso-seq called 198k genes, most of which we found to not be real genes. Thus, we chose to use the more conservative genome annotation that we present here, with the caveat that we may be missing some genes. In this annotation the total length of introns in the final assembly is 71.3Mb and total length of exons is 37.4Mb (Table 2).

**Table 1:** *Nanomia septata* genome assembly statistics.

Description	Haplotype 1 (alternate)	Haplotype 2 (primary)
Length	1794626896	1796533731
Contigs	5979.0	4964
Contig N50	888919	918025
Scaffolds	607	468
Scaffold N50	220372998.0	223932061.0
BUSCO complete	91.8	92.2
BUSCO single copy	85.4	85.4
BUSCO duplicated	6.4	6.8
BUSCO fragmented	2.8	2.8

**Table 2:** *Nanomia septata* annotation of haplotype 2 (primary).

Number of genes	26,323
Total gene length	110.4 MB
Total length of introns	71.3 MB
Total length of exons	37.4 MB

We manually curated the *Homeobox* and *Wnt* gene annotation, given their importance in cnidarian development (body and axis patterning) [23–26] and potential relevance to the unique colony-level growth of siphonophores. The original annotation had 38 of these genes and we found an additional 22 more using the Iso-sequencing of *Nanomia septata.* In total, *Nanomia septata* has 60 *homeobox* genes, split across 8 classes of genes (S1 Table). In comparison, other hydrozoans have on the order of 60−80 *homeobox* genes (S1 Table). More specifically, *Nanomia septata* has 17 *Antp* genes, including 4 *Hox-like* (*HOXL*) and 13 *NK-like* (*NKL*) members. Among *HoxL* genes, we identified one *Hox* gene (*Cnox-1*, located on chromosome 4), an unclassified *Hox-like* gene, and the two *paraHox* genes *Gsx* (*Cnox-2*) and Cdx (*Cnox-4*). We found 13 *Nk-like* genes, including *Nk-1*, *Nk-2* (three copies), and *Nk-6* genes (S1 Fig). In addition, the genome has nine *Wnt* genes identified as *Wnt* 1, 2, 3, 5 (two copies), 6, 7, 8 and A (S2 Fig). These have all been added so that in total, the annotation of haplotype 2 has 26,323 protein-coding genes (the first version had 26,301 genes).

### Differential gene expression across zooids

A previous bulk RNA-seq study assessed the differential expression of genes across different types of developing and mature zooids in 7 species using short read transcriptomes assembled with TRINITY as references [8]. We re-mapped the *Nanomia* RNA-seq reads used in that study to our new genome and annotation (six tissue/zooid types, some of which were split between developing and mature). These previous data are from specimens of the species now referred to as *Nanomia septata*, the same species that we sequence the genome of here. That manuscript was published before *Nanomia septata* was split from *Nanomia bijuga,* so they were referred to as *Nanomia bijuga* in this previous publication. Our remapping rates range from 32–51% for each RNA-seq sample, with a mean of 41%. The results from the original paper [8] are robust to the improved gene references. For example, female gonodendron and nectophores group together (S3 Fig). We included the male gonodendron dataset that was excluded in the original paper and found that it also closely groups with the female gonodendron and nectophores (S3 Fig), consistent with all these zooids being highly modified medusae that are retained within the colony. The pneumatophore, a specialized structure for buoyancy that is not a zooid, clusters closely with both gonodendra and nectophores (S3 Fig). Lastly, gastrozooids and palpons, which are both types of specialized polyps, have more similar expression to each other than to other zooids.

These re-mapped data also provide an updated list of genes with zooid-specific expression. We find that there is a total of 3,816 genes that have significant differential expression among zooid tissues and stages (S2 Table). Of these, 3,782 genes have expression specific to a single zooid type, mostly with increased expression in either mature female gonodendra or developing nectophores (S2 Table). Similar to the original study [8], genes with elevated expression in gastrozooids include many with functions related to digestion. With our new results, we also identify GOs (gene ontologies) for toxins that are abundant in female gonodendra (GO:0019835, GO:0030286, GO:0032991), nectophores (GO:0030286), palpons (GO:0004866) and the pneumatophore (GO:0004866) (S2 Table).

The remaining 34 out of 3,816 total genes have differential expression that is shared across multiple zooid types (S3 Table). Unsurprisingly, developing nectophores have shared differential expression with the mature pneumatophore and the mature female gonodendra, all of which cluster together in the PCA (S3 Table and S3 Fig). These shared differentially expressed genes have GOs for developmental processes such as morphogenesis of a branching structure and morphogenesis of epithelium. Specifically, TAGLN3, an actin gene, is shared and upregulated between the female gonodendra and pneumatophore, and UNC45A, a muscle development gene, is shared and upregulated between the female gonodendra and nectophore (S3 Table). Fox/forkhead genes have elevated expression in female gonodendra and developing nectophores, consistent with their role in medusa development observed in other cnidarians [27]. This updated list of differentially expressed genes in zooids will be useful for pursuing future in-depth zooid studies, including spatial expression experiments and single cell sequencing (S2 Table).

Additionally, we assessed the relationship between physical proximity of genes and the covariance of their expression across zooid types. The goal was to find genes that are close together in the genome and have similar expression across zooids. To do this we derived a new metric we call the expression proximity product (see methods). For each pair of genes, the expression proximity product is near zero if the genes are far apart or have low correlation of expression across zooids. They are near one if they are physically close and have high positive correlation of expression. They are near negative one if they are physically close and have strong negative correlation.

We found multiple regions in the genome with a high expression proximity product, i.e., regions that are enriched for genes with correlated expression across zooid types (S4 Fig 4). In all cases, these genes had elevated expression specific to male gonophores. These genes included spermatogenic genes (S4 Fig 4).

### Repeat analysis

**Fig 2:**
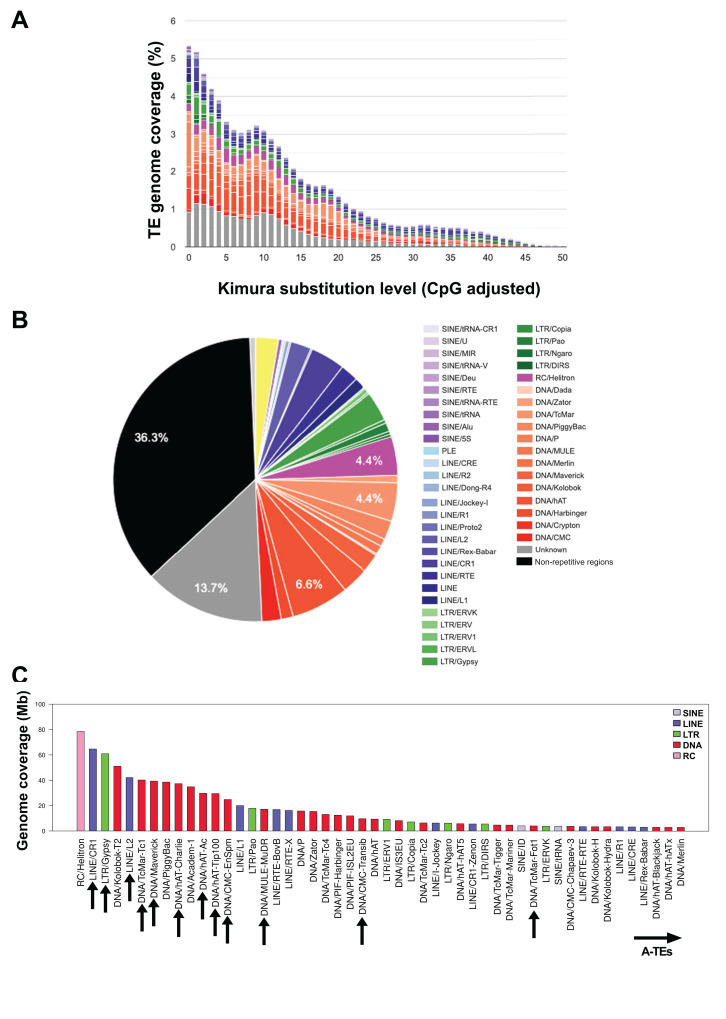
Repeat landscape of the *Nanomia septata* genome. A. Genome coverages and sequence divergences of TE families. The x-axis depicts the degree of Kimura substitution within repeat elements compared to consensus sequences, serving as an indicator of their relative age. The y-axis shows the genome coverage of each repeat family throughout the genome. Repeats with ancient activity are located towards the graph’s right, while those with more recent activity are found on the left side. B. Pie chart representing proportional representation of each TE family. Note that 36.3% of the genome consists of non-repetitive regions, while the remaining 63.7% are repetitive elements. C. Absolute genome coverage of top 50 TEs. Arrows indicate the eukaryotic Core-TEs. The eukaryotic Core-TEs collectively occupy 28.1% (505 Mb) of the *Nanomia septata* genome.

63.7% of the genome is composed of repetitive elements (Fig 2; S4 Table and S5 Table). This is consistent with previous estimates based on k-mers [21]. Notably, the proportion of DNA elements was high, with *TcMar-Tc1*, *Kolobok-T2*, *Maverick*, *PiggyBac*, and *hAT-Charlie* being abundant (Fig 2B). In addition, many other TEs are also abundant in the genome (Fig 2B,C; S5C Fig and S6 Fig; S4 Table). Furthermore, when examining the substitution levels of subfamilies of each transposon, we found a large number of subfamilies with low divergences (Fig 2A). This suggests that the *Nanomia septata* genome contains many young/active transposons. Additionally, other species belonging to *Nanomia* have been reported to exhibit a wide range of genome sizes [21].

Previously, Kon-Nanjo et al. [13] identified 14 highly conserved active TE families (A-TEs), mainly composed of DNA elements (*hAT-Ac*, *hAT-hATm*, *hAT-Tip100*, *hAT-Charlie*, *TcMar-Tc1*, *TcMar-Fot1*, *CMC-Transib*, *CMC-EnSpm*, *Sola-2*, *Maverick*, and *MULE-MuDR*), that are found across a wide range of eukaryotic genomes. In the *Nanomia septata* genome, these A-TEs collectively occupy 22.3% (401 Mb) of the genome (Fig 2C; S5 Fig 5). We also compared nine species of metazoans including two bilaterians (*Pecten maximus* and *Branchiostoma floridae*), seven cnidarians (*Nematostella vectensis*, *Acropora millepora*, *Rhopilema esculentum*, *Nanomia septata*, *Hydractinia symbiolongicarpus*, *Hydra viridissima*, and *Hydra vulgaris*). Among these species, *Nanomia septata* exhibited greater genome coverage by a wider variety of TE families, indicating a multi-TE family expansion (Cluster #1 in S5C Fig and S6 Fig). Additionally, 13.7% of repeats across the entire genome could not be classified, suggesting that they may be specific to *Nanomia septata* or siphonophores more broadly.

### Comparative cnidarian genomics

**Fig 3:**
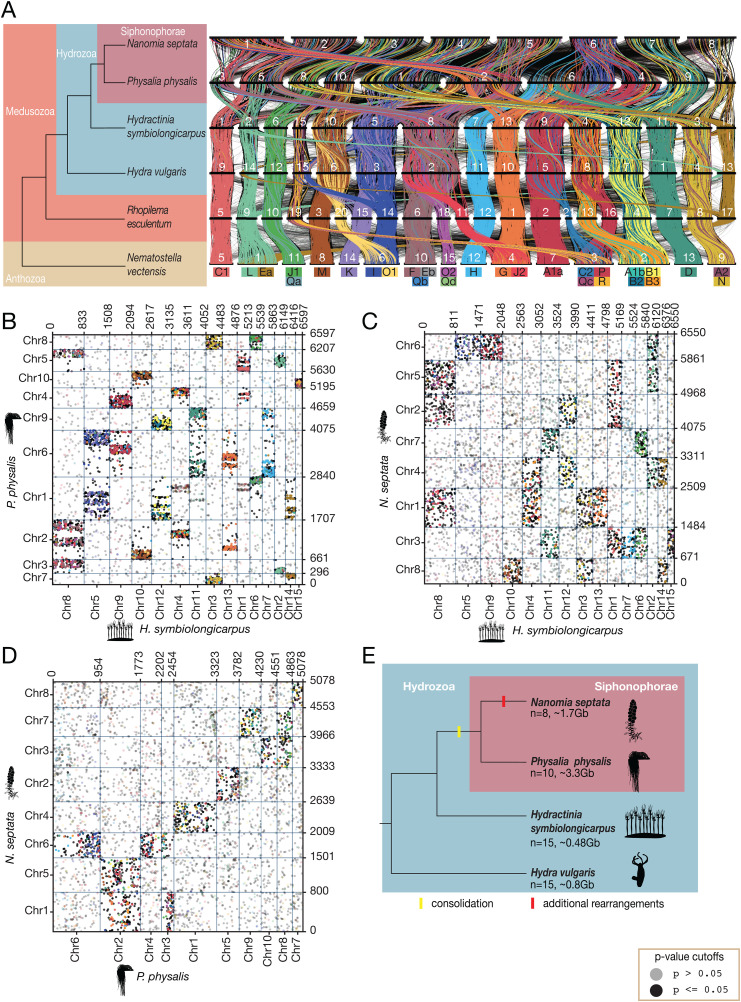
Syntenic transitions towards *Nanomia septata* karyotype. A. Ribbon plot of *Nanomia septata* and other cnidarians with chromosomes below. Chromosome labels with their accession numbers can be found in S7 Table. B-D. Oxford dot plots (ODP) of pairwise hydrozoan comparisons. B. ODP of *Hydractinia symbiolongicarpus* chromosomes vs *Physalia physalis* chromosomes. C. ODP of *Hydractinia symbiolongicarpus* chromosomes vs. *Nanomia sepatata* chromosomes. D. ODP of *Physalia physalis* chromosomes vs *Nanomia septata* chromosomes. E. Summary panel of siphonophores, *Nanomia septata* and *Physalia physalis*, and closely related hydrozoans that show where rearrangements, fusion and consolidation happened along the branches. Each protein ortholog is colored by the ancestral linkage group it was described in (BCnS [20]). Other protein orthologs are in black. Significance of linkage is denoted by a p-value (if significant opaque, otherwise transparent). P-value cutoffs are below panel E.

Many features of synteny are conserved across more than 500M years of cnidarian evolution, but are modified in the reduced karyotypes of Siphonophora (Fig 3A). A chromosome scale assembly of the siphonophore *Physalia physalis* was recently produced for a population analysis of *Physalia* [18] and is considered here. As there is detectable homology between n = 10 chromosomes in *Physalia physalis* (3 GB) and n = 8 chromosomes in *Nanomia septata* (1.7 GB), siphonophore genomes have shared ancestral chromosomal fusions. Differences between *Physalia physalis and Nanomia septata* reveal evolutionary changes within Siphonophora*.* While overall the chromosomal homologies are conserved there were still a few detectable fusions or translocations*.* These suggest that the reduced karyotype in *Nanomia septata* (n = 8) and *Physalia physalis* (n = 10) could have arisen by some fusions of the homologs of *Physalia physalis* chromosomes 2 and 3 to form LG 1, chromosomes 8 and 10 to form LG 3, and fusions of chromosome 6 and 4 to form LG 6 in *Nanomia septata* (Fig 3D). However, more translocations and mixing events could have occurred that are difficult to pin down with only two siphonophore genomes.

To investigate the origins of the siphonophore karyotype, we compared these genomes to their outgroups. Because *Hydra vulgaris* and *Hydractinia symbiolongicarpus* form a paraphyletic group with respect to siphonophores [19], their many shared features provide extensive information about the state at the start of the siphonophore stem branch (Fig 3A). These shared ancestral features include 15 chromosomes with highly conserved synteny (Fig 3A), and a typically small cnidarian genome size. Given the similarities between these two species, we make comparisons to *Hydractinia symbiolongicarpus* as a proxy for the ancestral state.

We compared the *Hydractinia symbiolongicarpus* and two siphonophore genomes, *Physalia physalis* and *Nanomia septata,* using oxford dot plots (Fig 3B-D). Comparison among these three species indicate that there is not a 1:1 correspondence between *Hydractina symbiolongicarpus* chromosomes (and other cnidarians) and any chromosomes in either siphonophore species. However, the *Physalia physalis* genome is more similar than *Nanomia septata* to *Hydractinia symbiolongicarpus*, showing many fusions of complete chromosomes and clear syntenic boundaries between them on *Physalia physalis* chromosomes. As such, there are discrete areas of homology between pieces of *Hydractinia symbiolongicarpus* chromosomes and pieces of *Physalia physalis* chromosomes, such as chromosome 15 in *Hydractinia symbiolongicarpus* which corresponds to chromosome 10 in *Physalia physalis* (Fig 3B). Only some inter-chromosomal translocations (e.g., *Physalia physalis* chromosome 6 and chromosome 9) were observed. This indicates a more ancestral state and a less mixed state of *Physalia physalis*. Furthermore, this shows that the genome of *Nanomia septata* has undergone more extensive reorganization losing syntenic identities through extensive mixing along the newly fused chromosomes (Fig 3C and 3D). The chromosomal mode of evolution in siphonophores thus corresponds to the “consolidation” process describing synteny loss primarily through extensive chromosomal fusion-with-mixing and karyotype reduction [28]. Supporting this, there is stronger statistical support for chromosomes between *Hydractina symbiolongicarpus* and *Physalia physalis* (S6 Table: Fig 3_Chr_Values_PanelB), while support values for chromosomes shared between *Hydractina symbiolongicarpus* and *Nanomia septata* are marginal (S6 Table: Fig 3_Chr_Values_PanelC). To understand the degree of scrambling of the previously described ancestral linkage groups (ALGs; bilaterian-cnidarian-sponge and unicellular-metazoan linkage groups), we visualize these ALGs in *Hydractinia symbiolongicarpus*, *Physalia physalis*, *Nanomia septata* (S7 Fig) which provides additional support for more extensive genome reorganization in *Nanomia septata*. All the supporting values for the pairwise analyses above are contained in S6 Table and chromosome labels in S7 Table.

Together, these findings suggest the role of chromosomal fusions, followed by mixing, as one of the major drivers in synteny loss in siphonophores (“consolidation” [28]) (Fig 3). Whether and how TE activity has contributed to within-chromosomal differences and mixing in *Physalia* compared to *Nanomia*, however, requires more chromosomal-scale species sampling to discern.

### Genomic diversity in *Nanomia*

To assess the broader context of diversity and population structure in *Nanomia*, we performed whole-genome short-read sequencing on 24 specimens collected over 19 years from the Atlantic Ocean (Virginia and Rhode Island), Pacific Ocean (Washington, California, Gulf of California, and Hawai’i), and Mediterranean Sea (Villefranche-sur-mer). Phylogenetic analyses of CO1 (S8 Fig) indicate that these specimens belong to *Nanomia septata* (7 samples from California, 3 from Washington), *Nanomia cara* (1 sample from Rhode Island), *Nanomia bijuga* (3 from Rhode Island, 2 from Hawai’i, 1 from Villefranche-sur-mer, 3 from Gulf of California)*,* and one undescribed species from Hawai’i consistent with *Nanomia* sp. 1 from Hosia et al. [7].

**Fig 4:**
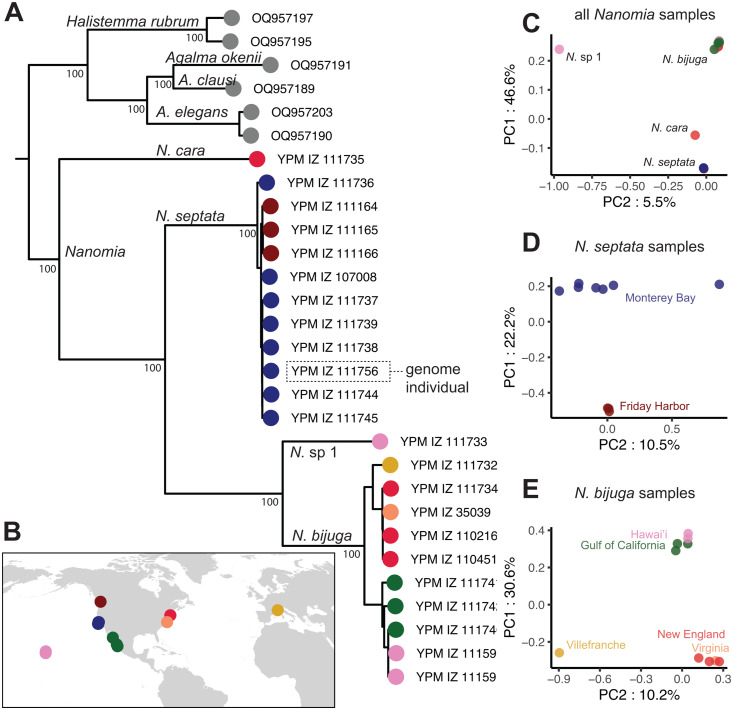
Population genomics of *Nanomia.* A. Phylogeny based on complete mitochondrial genomes. B. Map of collecting locations, which are coded by color. Colors in other panels correspond to locations here. C. PCA of all *Nanomia* specimens newly sequenced in this study. D. PCA of *Nanomia septata* specimens only. E. PCA of *Nanomia bijuga* samples only. All PCAs use the Nanomia *septata* reference genome. Analyses with species specific transcriptome references are shown in S10 Fig. Map made with Natural Earth (public domain) in R.

We assembled mitochondrial genomes from all sampled *Nanomia* and found them all to have the same conserved gene order recently reported for *Nanomia septata* and *Nanomia bijuga* [21]. The phylogenetic relationships of the full-length mitochondrial genomes of *Nanomia* (Fig 4A) are consistent with the relationships based on 16S and CO1 reported by Hosia et al. [7], with improved support (bootstrap values at all internal nodes 100), and with our CO1 analyses (S8 Fig). *Nanomia cara* is sister to all other *Nanomia,* and *Nanomia septata* is sister to a clade of *Nanomia bijuga* and *Nanomia* sp. 1 (Fig 4).

We also used k-mer distributions (S9 Fig) to confirm and extend recent observations of genome sizes of *Nanomia septata* and *Nanomia bijuga* [21]. New samples of *Nanomia septata* (n = 10, including the genome animal) have genome sizes between 1.4–1.8 GB and have similar repeat and heterozygosity statistics consistent with the single individual analyzed previously [21]. The single sample of *Nanomia cara* had a 1.7 GB genome, providing critical new information on a species for which no previous genome statistics were available. K-mer analyses of all *Nanomia bijuga* samples, as well as the specimen of *Nanomia* sp. 1 show that these species have much smaller genomes of about 0.7 GB and a lower fraction of repetitive elements. Taken together, these results indicate that there are two *Nanomia* species, *Nanomia septata* and *Nanomia cara*, with large genomes of 1.7 GB, and two, *Nanomia bijuga* and *Nanomia* sp. 1 with much smaller genomes that are about half the size at 0.7 GB. In combination with the phylogenetic results that show *Nanomia septata* sister to *Nanomia bijuga* and *Nanomia* sp. 1 and *Nanomia cara* sister to all of these species (Fig 4), these analyses suggest that the most recent common ancestor of *Nanomia* had a genome size of about 1.7 GB, and that there was then a large reduction in genome size along the branch that gave rise to *Nanomia bijuga* + *Nanomia* sp. 1 (Fig 4).

Combined population genomic analyses (see Methods), based on mapping each genome to the chromosome scale reference genome for *Nanomia septata*, clearly differentiate the species identified above (Fig 4C). There is also strong geographic structure to population diversity within species. Within *Nanomia septata* (Fig 4D), samples collected near Friday Harbor, Washington are distinct from those collected near Monterey, California. Within *Nanomia bijuga* (Fig 4E), we observe a separation between Pacific specimens and other specimens on PC1, and between the Mediterranean specimen and other specimens on PC2 in the graph (Fig 4E).

Reads from *Nanomia septata* map well to the *Nanomia septata* reference genome (85%–93% properly paired), but reads from *Nanomia cara* (44%), *Nanomia bijuga* (22–67%), and *Nanomia* sp. 1 (44%) map poorly (Fig 4). Previous work in *Physalia* found that results using transcriptome and genome references are highly similar [18]. To test whether results were robust to choice of reference sequence in *Nanomia*, we generated transcriptomes (Iso-seq) for *Nanomia bijuga* and *Nanomia septata* and used these as a reference for mapping reads from those species (S10 Fig). The PCAs of variation using species-specific transcriptome references are largely consistent with those derived when mapping to the reference genome (S10 Fig).

*Nanomia* species have distinct but overlapping ranges (Fig 4 and S10C Fig). For example, *Nanomia septata* is present across the North Pacific, from Japan, Alaska, and California. Likewise, *Nanomia bijuga* is found in parts of the Pacific, including Hawai’i, Japan, and Mexico, but also in the Atlantic and Mediterranean. Japan is a region of overlap among *Nanomia septata* and *Nanomia bijuga* and *Nanomia* sp. 1 ([7]). Additionally, *Nanomia* sp. 1 and *Nanomia bijuga* were both collected in Hawai’i, and *Nanomia cara* and *Nanomia bijuga* were both collected in Rhode Island.

## Discussion

A recent study found that most siphonophores, including *Nanomia septata*, have very large genomes relative to other cnidarians [21]. The chromosome-scale assembly of *Nanomia septata* presented here (Fig 1B) extends these findings to reveal that the genome of this siphonophore is highly derived in multiple respects, not just size. It has expanded repetitive elements in intergenic regions (Fig 2), as expected of a larger genome. There has also been a reduction from the 15 chromosomes seen in non-siphonophore hydrozoans to 8 chromosomes in *Nanomia septata*, a process that included extensive translocations and fusion-and-mixing, which has led to a highly reorganized genome in this species. Synteny analyses (Fig 3) with multiple cnidarian genomes, including an unannotated chromosome-scale reference genome from a population genomic analysis of the siphonophore *Physalia physalis* [18], reveal that there has been extensive genome reorganization along the stem branch that gave rise to the most recent common ancestor of siphonophores and then within siphonophores. Extensive genomic rearrangements could be a result of rapid chromosome evolution, as seen in other metazoans including clitellates, tunicates and now siphonophores [29–32].

The increased lability of siphonophore genomes relative to other hydrozoans could have a variety of causes, both neutral and adaptive. Demographic changes could lead to relaxed selection that results in genome expansion and reorganization [33]. Often, a high amount of heterogeneous repeats in other organisms are also a result of both accumulation and slow rates of removal of repetitive DNA [34]. Transposable element (TE) activity is a major contributor to genome expansion and the speed at which it happens, either through retroelement expansion [35, 36] or through putatively active TEs [13, 37]. Genome rearrangements can also be driven by deletions, insertions, translocations and inversions in chromosomes, all of which are a result of TE activity [38]. Additionally, particular changes in genome architecture could be associated with specific changes in the biology of siphonophores as has been similarly proposed in clitellates with the change from marine to freshwater [29–31]. However, the role of TE expansions in shaping cnidarian genomes has not revealed any particular correlate to rearrangement history; rather there are other principles and the impact of TE accumulation guiding chromosomal evolution [13, 37]. Irrespective of TE accumulation, it is possible that a karyotype reduction and genome rearrangements on the newly formed chromosomes in *Nanomia septata* could disrupt existing regulatory boundaries more easily, facilitating novel contact between cis-regulatory elements and developmental genes and the emergence of new developmental pathways [28]. The challenge is that siphonophores differ greatly from other animals, including their closest relatives, in a great number of ways including colonial development and a pelagic lifestyle. This makes it particularly challenging to associate specific genomic changes with changes in other aspects of their biology without additional data.

One of the most striking unique features of siphonophore biology is the extreme functional specialization between bodies (zooids) within siphonophore colonies, and the precise organization of these bodies into species-specific patterns. This unique morphology arises through unique developmental processes. Little is known, though, about the molecular mechanisms controlling these novel developmental processes. They could be completely novel to siphonophores, or the unique colony-level development of siphonophores could be coordinated with slight modifications to existing pathways. We examined two distinct features of genome organization that could be associated with developmental novelty. First, we used phylogenetic analyses of gene annotations to see if several known gene families central to cnidarian development (*homeobox, wnt,* etc.) have expanded in *Nanomia septata*. These gene families have not expanded and have around the same number of *homeobox* and *wnt* genes found in other closely related hydrozoans (S1 Table, S1 and S2 Fig). Second, we asked whether the extensive genome rearrangements found in *Nanomia septata* had brought genes that have similar expression across specialized zooids into closer physical proximity, perhaps for shared transcriptional regulation. Again, they have not (S4 Fig). The only exception are male gonophores, where we found genes relevant to spermatogenesis to be colocalized in multiple regions of the genome. This is not a siphonophore specific feature, though, and likely reflects the unique transcriptional demands of sperm as their genomes are packed [39–42]. As of now, the evolution of zooid specialization in siphonophore colonies is not caused by expansion of known gene families or by physical proximity of zooid specific genes. This leaves the relationship between the radically changed genomes of siphonophores and their radically unique morphology and development still to be determined. Further mechanistic developmental work in siphonophores will be critical to bridging this gap in understanding, an achievable goal with a new genome assembly and annotation.

Great strides have been made in siphonophore systematics in recent years, including a recent study of *Nanomia* ([7]. That work was based on morphology and phylogenetic analyses of two genes, and newly described the species our reference genome animal belongs to as *Nanomia septata*. Individuals of *Nanomia septata* had previously not been recognized as distinct from *Nanomia bijuga*. We sequenced 20 additional genomes of *Nanomia* and mapped them to the reference scale presented here (Fig 4). This corroborates the findings of Hosia et al. [7], adding support and new details, including regions of overlapping distribution of *Nanomia* species. *Nanomia bijuga* is found in multiple areas in the Atlantic, Mediterranean and Pacific. *Nanomia cara* overlaps with *Nanomia bijuga* in Rhode Island, while *Nanomia* sp. 1 overlaps with *Nanomia bijuga* in Hawai’i. These two regions harbor sympatric *Nanomia* species as there are no obvious oceanic barriers, though describing any specific species ranges is out of the scope of this project. These results are consistent with other recent studies [18, 43] that show that the diversity and geographic structure of gelatinous zooplankton has been greatly underestimated.

Understanding the specific features of global *Nanomia* diversity also has practical importance as *Nanomia septata* emerges as a model species for the study of development and other aspects of siphonophore biology. One of the most difficult parts of studying siphonophores is acquiring them since they must be regularly collected from the field. This makes it critical to know the diversity within the group, including how many species there are, where and when they are found, and how to tell them apart.

The genome biology of *Nanomia septata* presented here, including the chromosome-scale assembly along with its comparison to other cnidarian genomes and use as a reference for population studies, will facilitate future work on the evolution of functional specialization and transitions from one body to multiple bodies within a single organism.

## Methods

### Genome description

#### Specimen collection.

S8 Table presents sample data, including collection date, location, depth and Yale Peabody Museum accession number, for each newly sequenced specimen considered in this study and sequences from previous studies (labeled with which publication they are from). The chromosome-scale genome is from a single specimen, deposited at the Yale Peabody Museum as YPM-IZ-111756 (also known as D1399-SS10-NA-1-Nanomiabijuga), collected by ROV Doc Ricketts October 30, 2021 at 447 meters depth, latitude 36.7N, longitude 121.0672W in California. It was snap frozen by liquid nitrogen. All *Nanomia* specimens were collected in compliance with local, state and federal regulations. This study involved non-cephalopod, non-vertebrate specimens (Cnidaria: Siphonophora) so no IACUC or equivalent approval was needed. Specimens in Washington state were collected from Friday Harbor Laboratories (University of Washington, San Juan Island, WA) under institutional collecting authorization granted by the FHL Director. Rhode Island specimens were collected under the Rhode Island Division of Marine Fisheries (RIDMF) Scientific Collecting Permit under Casey Dunn. California and Hawaii specimens were collected collaboratively with the Monterey Bay Aquarium Research Institute (MBARI) in accordance with MBARI’s institutional research protocols and permit S-191140006-19114-001-02 from California Department of Fish and Wildlife issued to Steven Haddock. Hawaiian specimens were sampled in the open ocean (over 3 miles from shore) and no permit is necessary for offshore collecting. The Mediterranean specimen was collected by the Institut de la Mer de Villefranche (formerly Observatoire océanologique de Villefranche-sur-Mer).

### Karyotype

The karyotyping protocol was adapted from [44] with modifications as described below. We dissected the nectosomal and siphosomal growth zones (NGZ and SGZ) and basigasters (the base of gastrozooids closest to the stem and the origin point of cnidocytes) from four *Nanomia sp.* (*Nanomia* V4433 and *Nanomia* specimens 1, 2, and 3 from diffusion tube culture, labeled as is because no sampler number available) and placed them into small petri dishes (60 mm). Several basigasters were put into 1 hour colchicine treatment in the dark and the remaining basigasters, NGZ, and SGZ were put into a 5 hour colchicine treatment in the dark. Half of the basigasters from the 1 hour colchicine treatment were washed with DI water, by adding water in via drops and immediately pipetting it up. The rest of the basigasters in the 5 hour colchicine incubation were split up and washed with either a mix of DI water: filtered sea water (1:1) ratio or only DI water. Both were immediately pipetted up. We made Carnoy’s Fix fresh and added (3:1 methanol: glacial acetic acid) enough to cover the entire petri dish, which was then put on wet ice for 30 minutes.

Samples in petri dishes were transferred to slides and the liquid was removed. 20µl of 60% glacial acetic acid were added over the tissue, which was covered with siliconized coverslips using Sigmacote. We squashed preps manually using maximum pressure. Squashed preps were then placed overnight in a humidified chamber within a 4℃ refrigerator, and sealed with parafilm. The next day, siliconized coverslips were removed, samples were placed on dry ice, and each slide was washed with ~2mL 1X PBS to cover samples and then stained with DAPI 1:5000. Slides were imaged via confocal.

### Transcriptome sequencing

New transcriptomes were sequenced for two specimens, fresh tissue of *Nanomia septata* (Nanomia-culture-1) and frozen tissue of *Nanomia bijuga* (YPM-IZ-110217). We extracted RNA with the RNAqueous Total RNA Isolation Kit (Invitrogen) following the standard protocol as described in Church et al. [18]. We then removed DNA using the Turbo DNAse kit (Invitrogen).

Our samples were sent to Keck Microarray Shared Resource at Yale University for PacBio library prep and Iso-Sequencing, which is as follows. 400 ng of total RNA with a RIN of >7 was used as input. First-strand cDNA synthesis and PCR amplification of cDNA products were performed using the NEBNext Single Cell/Low Input cDNA Synthesis & Amplification Module (NEB kit), in conjunction with the Iso-Seq Express Template Switching Oligo and cDNA PCR Primer from the PacBio Iso-Seq Express Oligo Kit (101-737-500), following the PacBio Iso-Seq protocol. A minimum of 200 ng of amplified cDNA was used to prepare SMRTbell libraries with the SMRTbell Prep Kit 3.0 (102-396-000). The SMRTbell libraries were prepared according to the SMRT Link Sample Setup instructions, using the Polymerase Binding Sequel® II Binding Kit 3.1, and loaded onto the Sequel II for sequencing.

Delivered reads were put through our lab’s treeinform workflow as described in Church et al. [18] and at https://github.com/dunnlab/isoseq.

### Genome sequencing and initial assembly

Genome HiFi sequencing, Dovetail Omni-C proximity ligation, initial assembly, and annotation were completed by Cantata Bio on specimen YPM-IZ-111756 (sampling number D1399-SS10-NA-1).

Two combined DNA preps for the *Nanomia septata* sample were made, both of which used a CTAB protocol followed by the Qiagen mini kit. For the first prep, 180 mgs of sample incubated in 10 mls of CTAB, which yielded 8860ng of DNA. For the second prep, 500 mgs of sample incubated in 20 mls of CTAB, which yielded 8440 ng of DNA. DNA samples were quantified using Qubit 2.0 Fluorometer (Life Technologies, Carlsbad, CA, USA). The PacBio SMRTbell library (~20kb) for PacBio Sequel was constructed using SMRTbell Express Template Prep Kit 2.0 (PacBio, Menlo Park, CA, USA) using the manufacturer’s recommended protocol. The library was bound to polymerase using the Sequel II Binding Kit 2.0 (PacBio) and loaded onto PacBio Sequel II. Sequencing was performed on PacBio Sequel II 8M SMRT cells (2 were used). This produced 4,568,345 HiFi reads totalling 54.2GB in length.

PacBio CCS reads were assembled with Hifiasm1 v0.15.4-r347 using default parameters. Blast results of the Hifiasm output assembly against the nt database were used as input for blobtools2 v1.1.1 [45] and scaffolds identified as possible contamination were removed from the assembly. Finally, purge_dups3 v1.2.5 was used to remove haplotigs and contig overlaps [46].

### Structural annotation

Chromatin was fixed in place with formaldehyde in the nucleus. Fixed chromatin was digested with DNase I and then extracted, chromatin ends were repaired and ligated to a biotinylated bridge adapter followed by proximity ligation of adapter containing ends. After proximity ligation, crosslinks were reversed and the DNA purified. Purified DNA was treated to remove biotin that was not internal to ligated fragments. Sequencing libraries were generated using NEBNext Ultra enzymes and Illumina-compatible adapters. Biotin-containing fragments were isolated using streptavidin beads before PCR enrichment of each library. The Omni-C library was sequenced on an Illumina HiSeqX platform to produce ~ 30x sequence coverage.

The *de novo* HiFi assembly and Dovetail OmniC library reads were used as input data for HiRise, a software pipeline designed specifically for using proximity ligation data to scaffold genome assemblies [47,48]. Dovetail OmniC library sequences were aligned to the draft input assembly using bwa (https://github.com/lh3/bwa). The separations of Dovetail OmniC read pairs mapped within draft scaffolds were analyzed by HiRise to produce a likelihood model for genomic distance between read pairs, and the model was used to identify and break putative misjoins, to score prospective joins, and make joins above a threshold [49].

### Assembly refinement and Quality assessment

Juicebox (JBAT) [50] was used to manually curate and fix mis-assemblies and re-orient some contigs. We followed a similar pipeline used to manually curate the *Octopus vulgaris* genome [51], including using Chromap [52] to map the Omni-C data with a quality cutoff of Q0 and creating .hic files using 3d-dna [53] and artisanal (https://bitbucket.org/bredeson/artisanal) for use in Juicebox. In total, we went through 8 rounds of manual refinement of the genome. Scripts are deposited in our GitHub repository.

To review the quality of our assembly, we ran BUSCO metazoa_odb10 [54, 55] on our assembly and NCBI FCS.

### Gene annotation

Our gene annotation was completed by Cantata Bio as follows. Coding sequences from *Hydra vulgaris* (PRJNA703404), *Hydractinia symbiolongicarpus* (https://research.nhgri.nih.gov/hydractinia/), *Nematostella vectensis* (PRJNA667495) and *Rhopilema esculentum* (PRJNA523480 and PRJNA512552) were used to train the initial *ab initio* model for *Nanomia septata* using the AUGUSTUS software v2.5.5 [56]. Six rounds of prediction optimisation were done with the software package provided by AUGUSTUS [56]. The same coding sequences were also used to train a separate *ab initio* model for *Nanomia septata* using SNAP v2006-07–28 [57]. RNA-seq reads were mapped onto the genome using the STAR aligner software v2.7 [58] and intron hints generated with the bam2hints tools within the AUGUSTUS software. MAKER [59], SNAP [57] and AUGUSTUS [56] (with intron-exon boundary hints provided from RNA-seq) were then used to predict for genes in the repeat-masked reference genome. To help guide the prediction process, Swiss-Prot peptide sequences from the UniProt database [60] were downloaded and used in conjunction with the protein sequences from *Hydra vulgaris, Hydractinia symbiolongicarpus, Nematostella vectensis* and *Rhopilema esculentum* to generate peptide evidence in the Maker pipeline. Only genes that were predicted by both SNAP and AUGUSTUS softwares were retained in the final gene sets. To help assess the quality of the gene prediction, AED scores were generated for each of the predicted genes as part of the MAKER pipeline. Genes were further characterized for their putative function by performing a BLAST [61–63] search of the peptide sequences against the UniProt database. tRNAs were predicted using the software tRNAscan-SE v2.05 [64].

### Gene trees

We developed a Snakemake workflow to build gene trees using proteomes from *Nanomia septata*, two choanoflagellates, and 19 other animals (porifera, ctenophore, placozoa, ecdysozoa, spiralia, and chordata; listed in the github repo under: “analyses_annotation/gene_trees/config/download_targets.tsv”). The workflow downloads protein sequences, functionally annotates them using eggNOG-mapper v2.1.10 [65, 66] and infers individual gene trees using Orthofinder v2.5.4 [67, 68] with default parameters. It merges all gene trees in a master text file. We identified key genes that are conserved across Metazoa, with a focus on Cnidaria, including *Homeobox* and *Wnt* genes. We identified orthogroups containing *homeobox* sequences using human *homeobox* genes functionally annotated by eggNOG-mapper as landmarks. We corroborated *Nanomia septata* orthofinder results by identifying homeodomain-containing proteins running HMMER v3.4 (hmmer.org) with the hidden Markov model hd60.hmm from Zwarycz et al. [69], following Steinworth et al. [70]. In order to accurately identify *Wnt* and *Hox*-related genes, we used their gene trees from Orthofinder as a starting point for a more detailed phylogenetic analysis.

We retrieved 1,114 protein sequences from the orthogroup containing *Antp* homeobox genes and combined them with 111 previously identified *Hox*/*Hox*-related sequences from Khalturin et al. [71]. We aligned these 1,225 sequences with the L-INS-i algorithm using MAFFT v7 [72], which accounts for one conserved domain and long gaps. After manually trimming the variable regions and keeping only the 60 aa homeodomain, we added 270 *Hox*/*Hox*-related homeodomain sequences from Steinworth et al. [70] using MAFFT with the option –add fragments. We trimmed this alignment using trimAl (options -gt 0.9 -seqoverlap 90 -resoverlap 0.1) [73] and inferred a maximum likelihood tree using IQTree2 [74] with the evolutionary model LG + R8, selected by ModelFinder [75]. Ultrafast bootstrap branch support values were calculated based on 1,000 replicates. This tree was rooted on the stem branch of Nk-like genes.

For inferring the *Wnt* gene tree, we first combined all protein sequences assigned to *Wnt* orthogroups (325 sequences), 13 *Wnt* sequences from the hydrozoan jellyfish *Clytia hemisphaerica* previously identified by Condamine et al. [76], and 12 *Wnt* sequences from the cubozoan jellyfish *Tripedalia* identified by Khalturin et al. [76]. We removed two sequences from *Orbicella faveolata* that were exceptionally long (XP_020618804.1 and XP_020632229.1) and one sequence from *Trichoplax adhaerens* that was extremely short (TriadP8072). We aligned the remaining 347 sequences and 7 DASH sequences (for structural homology, see [77] using the FFT-INS-ii algorithm on MAFFT v7 [72]. Using IQTree2 [74], we selected the best evolutionary model (LG + R9) and inferred a maximum likelihood tree, with ultrafast bootstrap branch support values based on 1,000 replicates and midpoint rooted.

Additional *Homeobox* genes that we found manually (not originally included in the annotation) have been added to annotation files (transcript, protein and gff). These sequences were found in the *Nanomia septata* Iso-sequencing and were checked before their addition.

### Differential expression and zooid covariance/co-expression analyses

We re-examined previously published zooid-specific bulk RNA-seq data [8] using salmon v1.10.1 [78]. Counts were then imported with tximport v1.26.1 into DESeq2 v1.38.3. Our scripts also produced figures to show the variance/comparison between different zooid types and developmental stages and how they relate to one another (S3 Fig).

We next sought to identify any genes that are close to each other physically and have strong expression covariance across zooids. This analysis was implemented in the script in the repository https://github.com/dunnlab/nanomia_genome/blob/main/analyses_de/expression-proximity.ipynb. First, for each gene we average the normalized counts across replicates for each combination of tissue and stage that was sequenced. This generated a dataframe with one row per gene and one column per tissue and stage. We ordered genes by chromosome and start site. The gene expression covariance matrix was calculated based on the averaged normalized expression values. We then created a physical distance matrix between genes, where the distance is nan if the genes are on different chromosomes and the number of genes away if they are on the same chromosome, e.g., 0 for self, 1 for adjacent, etc. We then derived a proximity matrix from this distance matrix by passing it through a Gaussian kernel and taking the inverse. In the proximity matrix, the value is 0 if the genes are on different chromosomes, 1 on the diagonal (i.e., the proximity of a gene to itself is 1), and has a smooth transition from 1 to 0 as the genes become more distant. The standard deviation of the kernel was set such that proximity is 0.5 for genes that have a distance of 50. Finally, we took the element-wise product of the expression covariance matrix and the proximity matrix, producing a matrix we refer to as the expression proximity product. Values in this expression proximity product are near zero if the genes are far apart or have low correlation. They are near one if they are physically close and have high positive correlation, and near negative one if they are physically close and have strong negative correlation.

### Custom repetitive element library generation and TE detection

Since the *Nanomia septata* genome presented here is the first genome assembly for the genus *Nanomia*, the number of repetitive elements registered in databases, such as Dfam, is limited relative to typical model animals [79]. Thus, we generated a custom repeat library using RepeatModeler v2.0.5 [79, 80]. Inactivated TEs accumulate mutations, including base substitutions, insertions, and deletions [81, 82]. Because of this, when RepeatClassifier, a submodule of RepeatModeler, performs a search against Dfam using rmblast, the number of hits can be extremely low, resulting in a library containing many uncharacterized sequences (“Unknown” repeats). This is particularly evident for non-model species whose repetitive elements are not well-represented in Dfam. To address this challenge, we used an approach similar to that used in our previous studies [13, 80]. First, we generated a custom repetitive element library using RepeatModeler v2.0.5 [80] with the *Nanomia septata* genome assembly as the query. Then, we obtained all Dfam HMM profiles of TEs from the Dfam database, version 3.8 (https://dfam.org/home). Because the Dfam HMM profile dataset for TEs is a huge data size, we divided these HMM profiles into smaller chunks, each containing 25,000 HMM profiles. Each chunk of HMM profiles was indexed using hmmpress [83,84]. Then, we performed an HMM-based search against the Dfam HMM profiles using nhmmscan with default parameters, using the custom repeat library as the query [83,84]. For each query sequence, unclassified query sequences were assigned names using the annotation of the hit with the lowest E-value (≤ 0.05). Using the updated custom repetitive element library, we conducted RepeatMasker [85] on the *Nanomia septata* genome assembly with the options of -parallel 70 -gff -a -dir -xsmall. For other species, we obtained genome assemblies from the NCBI genome database, and performed RepeatModeler [80] and RepeatMasker [85] in the same manner.

### Comparative cnidarian genomics

#### Synteny.

We performed synteny analysis using odp v0.3.3 [20, 86] on the hap2 *Nanomia septata* genome against other publicly available cnidarian chromosome-scale genomes with a proteome and available annotation (*Nematostella vectensis* GCF_932526225.1, *Rhopilema esculentum* [87] which was formatted by Schultz et al. [86], *Hydra vulgaris* GCA_022113875.1, and *Hydractinia symbiolongicarpus* GCF_029227915.1, and *Physalia physalis* [18] (S7 Table). The *P. physalis* genome was annotated using the *P. physalis* Iso-seq data [18], SRR29478154), and processed with the Dunn Lab Iso-seq workflow (https://github.com/dunnlab/isoseq, modified from Guang et al. [88]. We selected the transcriptome with a cutoff value of 8 under strict conditions, resulting in 13,048 transcripts. The transcriptome was then mapped to the genome using minimap2 v2.2 [89, 90], and the resulting *bam* file was sorted using SAMtools v1.18 [91]. The *bam* file was then converted to a *gff* file using spliced_bam2gff v1.3 [92]. The input proteome was generated by translating the mapped transcriptome using TransDecoder v5.71 (https://github.com/TransDecoder/TransDecoder). We then plotted syntenic relationships using the main odp v0.3.3 workflow between each pair of species and identified ancestral linkage groups as defined in Simakov et al. [20] and Schultz et al. [86]. We ran odp_rbh_to_ribbon on *.rbh* files containing information on bilaterian-cnidarian-sponge (BCnS) ancestral linkage groups [86] to generate a ribbon plot.

### Population genetics and mitochondrial genomes

*Nanomia* samples (sample identifiers NA28–38 and WS2–10) were sequenced via Illumina NovaSeq S4 2X150 bp PE. Coverage ranged from 250 million reads to 900 million reads (S11 Fig). Genome size was estimated for samples NA28–38 and WS1–10, for which there was sufficient coverage to achieve an accurate estimate. For this analysis, we used a Snakemake workflow [93] to count k-mers using Jellyfish v2.3.0 [94] and Genomescope v2.0 [95, 96] as detailed in Ahuja et al. [21].

For all samples, libraries were trimmed for Illumina adapters using Trimmomatic v0.39 [97] and library quality was assessed using FastQC v.0.11.9 [98]. Mitochondrial genomes were assembled from a subset of 10,000,000 read pairs using GetOrganelle v.1.7.7.0 [99], using as a seed the SRR23143273 and SRR23143286 publicly available mitochondrial (mt) genomes from *Nanomia bijuga* sample CWD116 and *Nanomia* sp. California NA19 [21], and using the animal_mt database and default parameters. All mitochondrial genomes were assembled as linear, with the exception of YPM-IZ-35039; upon visual inspection of mitogenome alignment, this was considered to be a misassembly, and we manually corrected this genome. The mitogenome for YPM-IZ-107008 was assembled in three fragments. For downstream analyses the largest fragment was used. Mitochondrial genomes were annotated with MITOS2 [100] and tRNAscan [64].

A mitochondrial genome was also assembled from PacBio HiFi reads for the genome reference specimen by blasting CO1 from the 2 seed *Nanomia* mt genomes and identifying the appropriate contigs. Assembled mitochondrial sequences were combined with publicly available mt genomes for *Nanomia* (OQ957219.1, OQ957196.1), *Halistemma* (OQ957197.1, OQ957195.1), and *Agalma* (OQ957203.1, OQ957191.1, OQ957190.1, OQ957189.1), and then aligned using MAFFT, --adjustdirectionaccurately option v.7.505 [72] and a phylogeny was inferred using IQTree2 software [74], model autoselected [75] and 1,000 ultrafast bootstraps [101]. Assembled mitochondrial sequences are available on our GitHub repository at https://github.com/dunnlab/nanomia_genome, which is also archived at https://doi.org/10.5281/zenodo.19629162.

In addition, individual marker sequences for the mitochondrial cytochrome oxidase I (CO1) gene were assembled from a subset of 10 million reads using *in silico* PCR as implemented in sharkmer (available at https://github.com/caseywdunn/sharkmer). Sequences were combined with publicly available *Nanomia* sequences, as published in Hosia et al. [7], along with sequences for *Halistemma* and *Agalma* from NCBI. Sequences were aligned with MAFFT, and gene trees inferred with IQTree2, as described above. Assembled CO1 sequences are available on our GitHub repository.

Population structure was analyzed following the workflow described in Church et al. [18]. In brief: libraries were assessed for contamination with Kraken2 [102]; sample kinship was evaluated using Plink2 [103]; reads were then mapped to the reference assembly using BWA [104] and alleles were called using BCFTools v1.16 [105]; genotype likelihoods were calculated using ANGSD v.0.935 [106]; principal components of genomic variation were analyzed on genotype likelihoods using PCANGSD v1.21 [107]. We repeated this analysis for *Nanomia bijuga* and *Nanomia septata* separately, using assembled transcriptomes from PacBio Iso-Seq data as the reference, and compared our results to those using the *Nanomia septata* reference genome.

## Supporting information

S1 Table Number of sequences placed in orthogroups containing *homeobox* genes. These numbers refer to homeodomain containing proteins placed in orthogroups identified by searching for landmark human homeobox sequences functionally annotated by EggNOG-mapper (Cantalapiedra et al. 2021; Huerta-Cepas et al. 2019). We corroborated *N. septata* orthofinder results by identifying homeodomain-containing proteins with HMMER (hmmer.org), confirming they were placed in verified orthogroups and searching for highly divergent homeodomain-containing proteins that could have been placed in separate orthogroups. These numbers do not include sequences that were unassigned to an orthogroup or not annotated in the genome of these species. Classification of genes according to Holland et al. (2007). Orthogroup gene trees sorted by *homeobox* class available in the supplementary data folder. *N. septata*: *Nanomia septata*, *A. queenslandica*: *Amphimedon queenslandica*, *N. vectensis*: *Nematostella vectensis*, *M. virulenta*: *Morbakka virulenta*, *R. esculentum*: *Rhopilema esculentum*, *H. vulgaris*: *Hydra vulgaris*, *C. hemisphaerica*: *Clytia hemisphaerica*, *H. symbiolongicarpus*: *Hydractinia symbiolongicarpus*.
(XLSX)

S2 Table Differentially expressed genes for all zooid types and stages in *Nanomia septata.* (XLSX)

S3 Table Differentially expressed genes that are shared between some zooid types in *Nanomia septata.* (XLSX)

S4 Table Genome coverage of major TE groups. (XLSX)

S5 Table Genome coverage of TE families. (XLSX)

S6 Table Support values for synteny analyses for Fig 3 and S7 Fig. (XLSX)

S7 Table The names used for chromosomes in the manuscript vs. their accession number/sequence name. (XLSX)

S8 Table Specimen data and accession numbers of all WGS *Nanomia* samples that are introduced in this study and samples from previous studies, including CWD16 and NA19 from Ahuja et al. 2024 and zooid RNA-seq datasets from Munro et al. 2022. (XLSX)

S1 Fig *Antp* homeobox gene tree (*Hox* and *Nk-related* gene families). Maximum likelihood tree of 1,465 *Hox* and *Nk-related* sequences, built under model LG + R8, with ultrafast bootstrap branch support (1,000 replicates) values along branches. This tree contains protein sequences from the orthogroup with *Antp* homeobox genes, in addition to previously identified *Hox*/*Hox*-related sequences from Khalturin et al. (2019) and Steinworth et al. (2022). *Nanomia septata* sequences are colored in magenta and highlighted with a star. Sequence names retrieved from *Antp* orthogroup contain the species name (followed by _pep), the sequence ID from original source file and a label with the functional annotation generated by eggNOG-mapper. Branch lengths were transformed to equal for display.
(PDF)

S2 Fig *Wnt* gene tree. Maximum likelihood phylogenetic tree, midpoint rooted, inferred using the LG + R9 substitution model from 347 protein sequences gathered from representatives of Cnidaria and Bilateria. *Nanomia septata* sequences are colored in magenta. Sequence names contain the species name (followed by _pep), the sequence ID from original source file and a label with the functional annotation generated by eggNOG-mapper. Condamine (2019) Wnt sequences from *Clytia* are labeled CheWnt1–11. *Wnt* sequences from *Tripedalia cystophora* previously identified by Khalturin et al. (2019) are included and contain “’tri_comp”. Ultrafast bootstrap values are displayed by their corresponding nodes. Scale bar denotes number of substitutions per site. Original tree is available in the supplementary data folder.
(PDF)

S3 Fig Differentially expressed gene analysis for *Nanomia septata.* Transcriptomes from distinct zooid types and pneumatophores of *Nanomia septata* at different developmental stages cluster according to their transcriptional similarity.
(EPS)

S4 Fig Expression proximity product for genes on each *Nanomia septata* chromosome. Each panel presents a chromosome. Each bar is a gene, and the x-axis indicates the index of the gene. Color indicates how strongly the expression of a gene covaries with its neighbors (i.e., the more yellow a gene is, the more similar its expression is across zooids to neighboring genes).
(EPS)

S5 Fig Transposable Element (TE) expansions in the *Nanomia septata* genome. A. Size distribution of the cnidarian genome assemblies available in the NCBI Genome database. B. Number of chromosomes in the genome assemblies available in the NCBI Genome database. C. Relative genome coverage of TE families in cnidarian genomes and two bilaterian outgroup species. For each TE family, genome coverage in each species was calculated and was normalized (z-transformed) by row (across species). The dendrogram at the left of the heatmap represents the result of hierarchical clustering performed using the Euclidean distance metric and the complete linkage method. The cluster #1 is composed of TE families which are overrepresented in *N anomia* compared to other species. Black arrows indicate 14 TE families belonging to the A-TEs. Note that in addition to the TE families increasing in *Nanomia septata,* there are TE families showing clade-specific expansion in other animals as well. Abbreviations: PMA, *Pecten maximus*; BFL, *Branchiostoma floridae*; NVE, *Nematostella vectensis*; AMI, *Acropora millepora*; RES, *Rhopilema esculentum*; NAN, *Nanomia septata*; HSY, *Hydractinia symbiolongicarpus*; HVI, *Hydra viridissima*; HVU, *Hydra vulgaris*.
(EPS)

S6 Fig Genome coverages of TEs. A. Genome coverages of SINE, LINE, LTR, DNA, and Rolling-circle elements. The y-axis indicates genome coverage of TEs (Gb). B. Genome coverages of the eukaryotic core TEs. The y-axis indicates genome coverage of TEs (Mb) and x-axis indicates species. The color scheme is the same as in panel a. Abbreviations: PMA, *Pecten maximus*; BFL, *Branchiostoma floridae*; NVE, *Nematostella vectensis*; AMI, *Acropora millepora*; RES, *Rhopilema esculentum*; NAN, *Nanomia septata*; HSY, *Hydractinia symbiolongicarpus*; HVI, *Hydra viridissima*; HVU, *Hydra vulgaris*.
(EPS)

S7 Fig BCnS ALGs and unicellular-metazoan (pre-metazoan) ALGs in *Hydractinia symbiolongicarpus, Nanomia septata and Physalia physalis* shown via Oxford dot plots (ODP). A. ODP of ancestral linkage groups from Simakov et al. (2022) vs. *Hydractinia symbiolongicarpus* chromosomes. B. ODP of ancestral linkage groups from Schultz et al. (2023) vs. *Hydractinia symbiolongicarpus* chromosomes. C. ODP of ancestral linkage groups from Simakov et al. (2022) vs. *Nanomia septata* chromosomes. D. ODP of ancestral linkage groups from Schultz et al. (2023) vs. *Nanomia septata* chromosomes. E. ODP of ancestral linkage groups from Simakov et al. (2022) vs. *Physalia physalis.* F. ODP of ancestral linkage groups from Schultz et al. (2023) vs. *Physalia physalis.*
(PNG)

S8 Fig CO1 *Nanomia* phylogram. Includes CO1 sequences from *Nanomia* genomes presented in the paper as well as CO1 sequences from NCBI.
(PNG)

S9 Fig Genomescope fit models for 1 representative *Nanomia* sample per group in the phylogeny, including *Nanomia septata, Nanomia bijuga, Nanomia cara* and *Nanomia* sp. 1. (EPS)

S10 Fig *Nanomia* specimens mapped using two separate transcriptome references to look at variance. A. *Nanomia septata* samples mapped against *Nanomia septata* Iso-seq transcriptome reference, displaying two distinct clusters, one cluster from Washington and the other from California. B. *Nanomia bijuga* samples mapped against *Nanomia bijuga* Iso-seq transcriptome reference showing samples falling into multiple distinct clusters, including a cluster of Atlantic specimens + Mediterranean, a cluster of Hawaiian+ GoC and 2 GoC samples that are more varied than the others. C. Map of all *Nanomia* specimen collection spots colored by population. Map made with Natural Earth (public domain) using R with packages sf (v1.1) and rnaturalearth (v4.1).
(PNG)

S11 Fig *Nanomia* sample coverage statistics. A. The percent reads of *Nanomia* samples that are not classified as contamination, all falling >80%. B. The total reads for each *Nanomia* specimen. C. The total number of mapped reads against the *Nanomia septata* genome.
(PNG)
