# Supplementary figures and images for: Siphonophore genome structure and the evolution of functional specialization
Source: PLoS One. 2026 Jul 2;21(7):e0351247. doi: 10.1371/journal.pone.0351247 (PMC13327316; doi:10.1371/journal.pone.0351247)

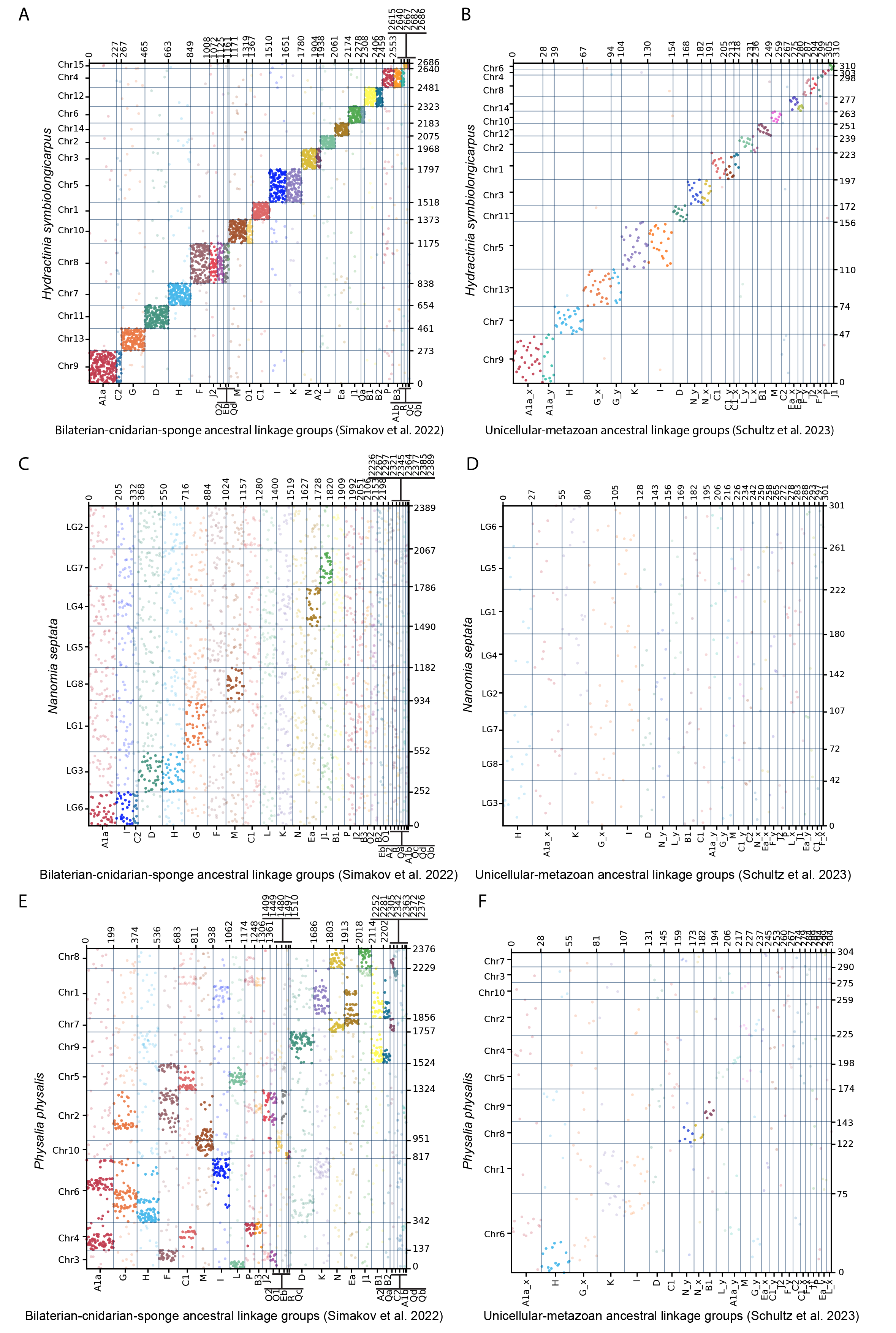

Supplement: S7 Fig — A. ODP of ancestral linkage groups from Simakov et al. (2022) vs. Hydractinia symbiolongicarpus chromosomes. B. ODP of ancestral linkage groups from Schultz et al. (2023) vs. Hydractinia symbiolongicarpus chromosomes. C. ODP of ancestral linkage groups from Simakov et al. (2022) vs. Nanomia septata chromosomes. D. ODP of ancestral linkage groups from Schultz et al. (2023) vs. Nanomia septata chromosomes. E. ODP of ancestral linkage groups from Simakov et al. (2022) vs. Physalia physalis. F. ODP of ancestral linkage groups from Schultz et al. (2023) vs. Physalia physalis. (PNG) [file pone.0351247.s015.png]

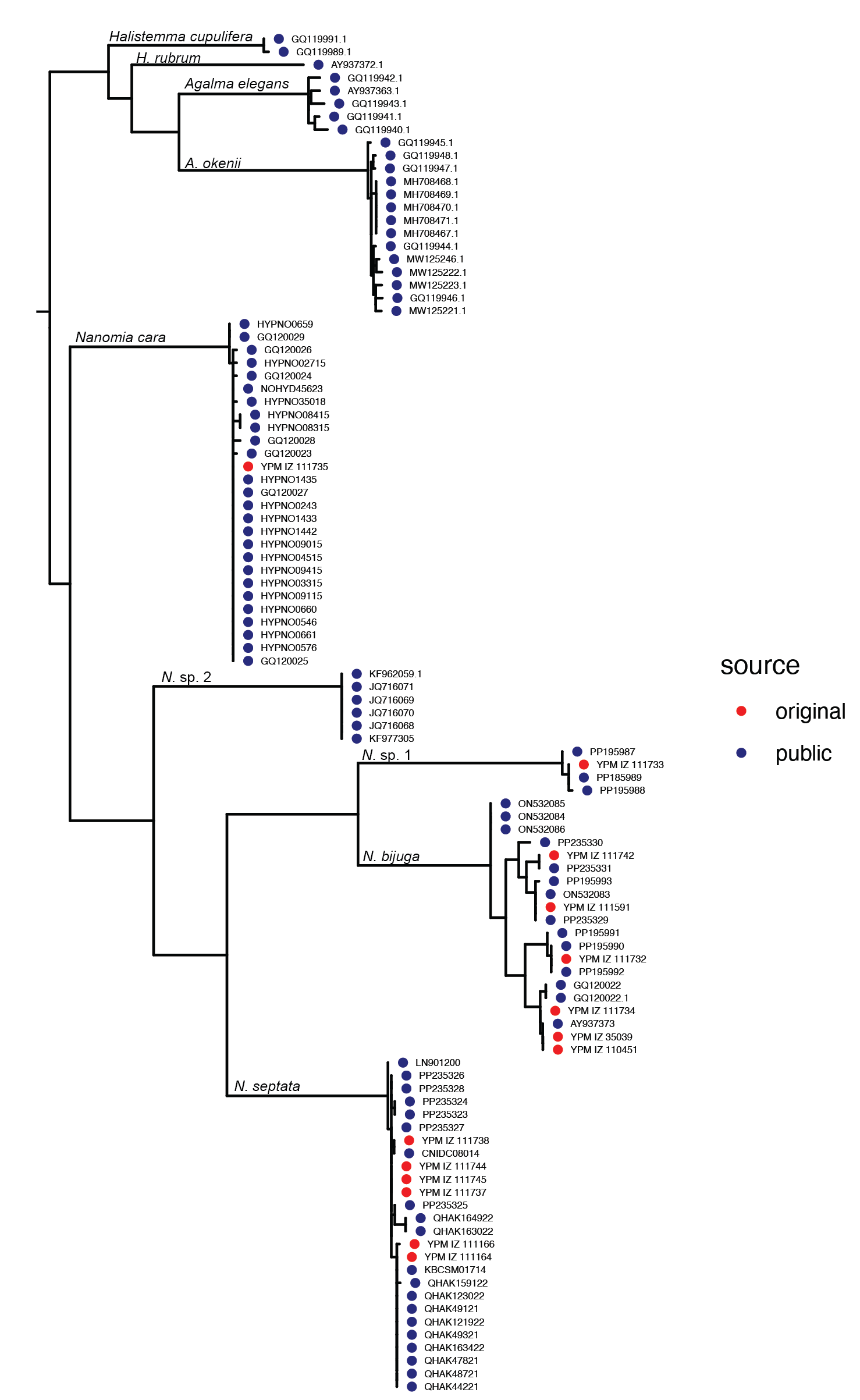

Supplement: S8 Fig — Includes CO1 sequences from Nanomia genomes presented in the paper as well as CO1 sequences from NCBI. (PNG) [file pone.0351247.s016.png]

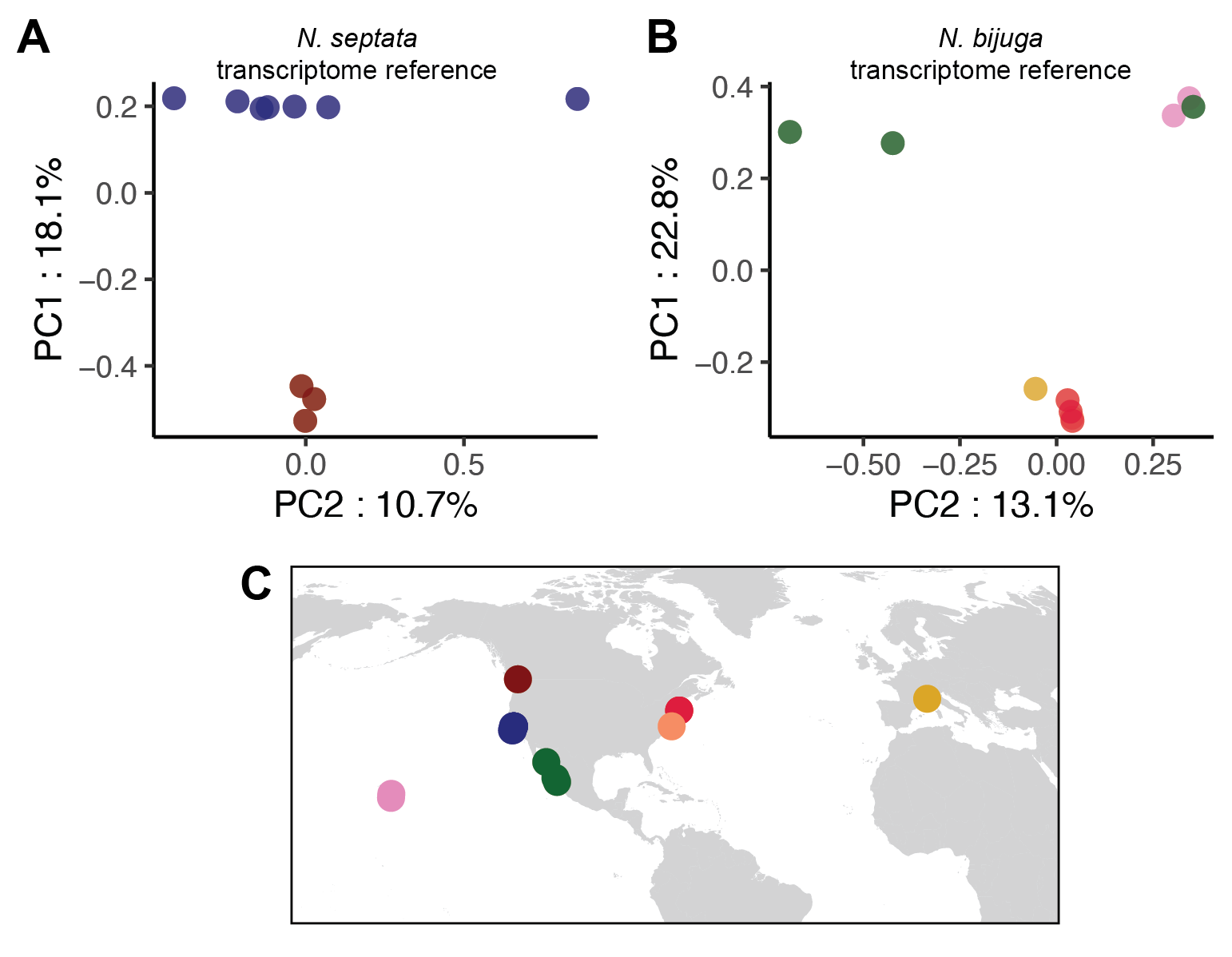

Supplement: S10 Fig — A. Nanomia septata samples mapped against Nanomia septata Iso-seq transcriptome reference, displaying two distinct clusters, one cluster from Washington and the other from California. B. Nanomia bijuga samples mapped against Nanomia bijuga Iso-seq transcriptome reference showing samples falling into multiple distinct clusters, including a cluster of Atlantic specimens + Mediterranean, a cluster of Hawaiian+ GoC and 2 GoC samples that are more varied than the others. C. Map of all Nanomia specimen collection spots colored by population. Map made with Natural Earth (public domain) using R with packages sf (v1.1) and rnaturalearth (v4.1). (PNG) [file pone.0351247.s018.png]

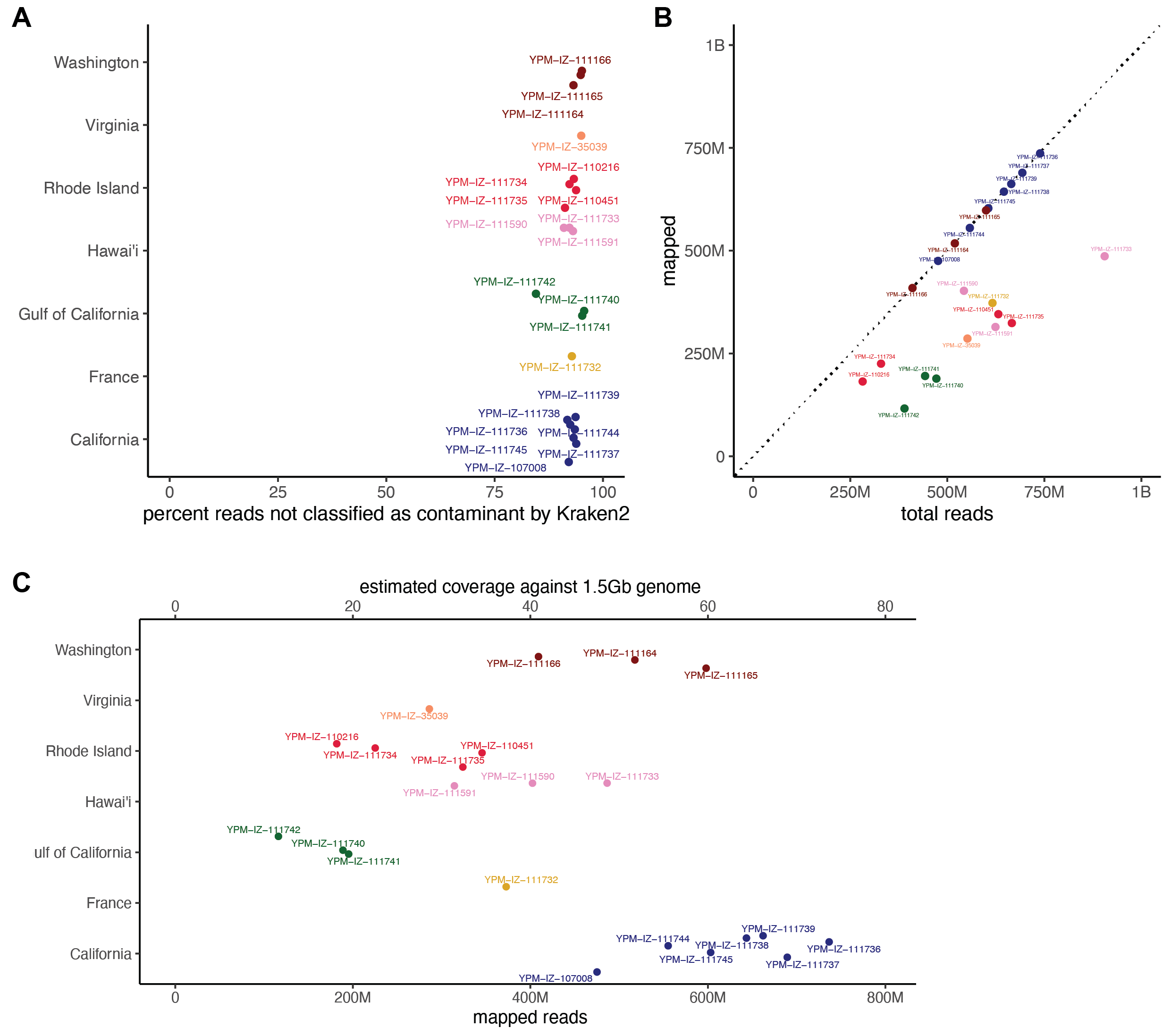

Supplement: S11 Fig — A. The percent reads of Nanomia samples that are not classified as contamination, all falling >80%. B. The total reads for each Nanomia specimen. C. The total number of mapped reads against the Nanomia septata genome. (PNG) [file pone.0351247.s019.png]
