# Supplementary material for: Siphonophore genome structure and the evolution of functional specialization
Source: PLoS One. 2026 Jul 2;21(7):e0351247. doi: 10.1371/journal.pone.0351247 (PMC13327316; doi:10.1371/journal.pone.0351247)
Supplement: S1 Fig — Maximum likelihood tree of 1,465 Hox and Nk-related sequences, built under model LG + R8, with ultrafast bootstrap branch support (1,000 replicates) values along branches. This tree contains protein sequences from the orthogroup with Antp homeobox genes, in addition to previously identified Hox/Hox-related sequences from Khalturin et al. (2019) and Steinworth et al. (2022). Nanomia septata sequences are colored in magenta and highlighted with a star. Sequence names retrieved from Antp orthogroup contain the species name (followed by _pep), the sequence ID from original source file and a label with the functional annotation generated by eggNOG-mapper. Branch lengths were transformed to equal for display. (PDF) [file pone.0351247.s009.pdf]

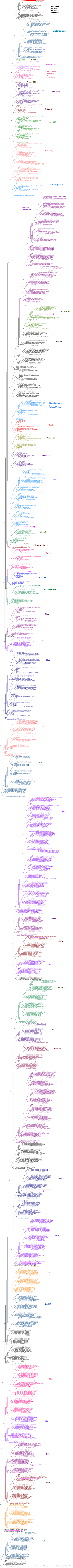

Supplementary Figure 1. Antp homeobox gene tree (Hox and Nk-related gene families). Maximum likelihood tree of 1,465 Hox and Nk-related sequences, built under model LG+R8, with ultrafast bootstrap branch support (1,000 replicates) values along branches. This tree contains protein sequences from the orthogroup with Antp homeobox genes, in addition to previously identified Hox/Hox-related sequences from Khalturin *et al*. (2019) and Steinworth *et al*. (2022). *Nanomia septata* sequences are colored in magenta and highlighted with a star. Sequence names retrieved from Antp orthogroup contain the species name (followed by _pep), the sequence ID from original source file and a label with the functional annotation generated by eggNOGG-mapper. Branch lengths were transformed to equal for display. Original tree is available in the supplementary data folder.
