# Supplementary material for: Siphonophore genome structure and the evolution of functional specialization
Source: PLoS One. 2026 Jul 2;21(7):e0351247. doi: 10.1371/journal.pone.0351247 (PMC13327316; doi:10.1371/journal.pone.0351247)
Supplement: S2 Fig — Maximum likelihood phylogenetic tree, midpoint rooted, inferred using the LG + R9 substitution model from 347 protein sequences gathered from representatives of Cnidaria and Bilateria. Nanomia septata sequences are colored in magenta. Sequence names contain the species name (followed by _pep), the sequence ID from original source file and a label with the functional annotation generated by eggNOG-mapper. Condamine (2019) Wnt sequences from Clytia are labeled CheWnt1–11. Wnt sequences from Tripedalia cystophora previously identified by Khalturin et al. (2019) are included and contain “’tri_comp”. Ultrafast bootstrap values are displayed by their corresponding nodes. Scale bar denotes number of substitutions per site. Original tree is available in the supplementary data folder. (PDF) [file pone.0351247.s010.pdf]

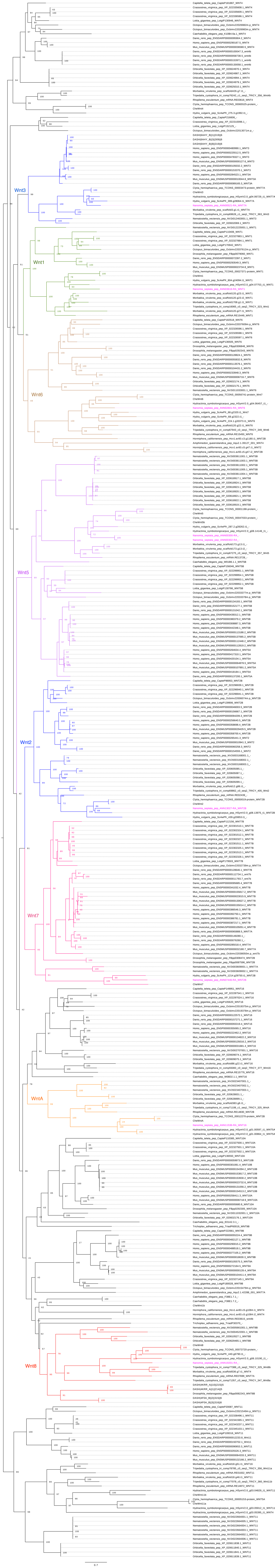
Wnt3
Wnt1
Wnt6
Wnt5
Wnt2
Wnt7
WntA
Wnt8
